# Decentralized Policy-Hidden Fine-Grained Redaction in Blockchain-Based IoT Systems

**DOI:** 10.3390/s23167105

**Published:** 2023-08-11

**Authors:** Hongchen Guo, Xiaolong Tao, Mingyang Zhao, Tong Wu, Chuan Zhang, Jingfeng Xue, Liehuang Zhu

**Affiliations:** 1 School of Computer Science and Technology, Beijing Institute of Technology, Beijing 100081, China; guohongchen@bit.edu.cn (H.G.); xuejf@bit.edu.cn (J.X.); 2 School of Cyberspace Science and Technology, Beijing Institute of Technology, Beijing 100081, China; ac_xiaolongt@163.com (X.T.) mingyangz@bit.edu.cn (M.Z.); chuanz@bit.edu.cn (C.Z.); liehuangz@bit.edu.cn (L.Z.)

**Keywords:** blockchain-based IoT systems, fine-grained redaction, policy hiding, decentralization

## Abstract

Currently, decentralized redactable blockchains have been widely applied in IoT systems for secure and controllable data management. Unfortunately, existing works ignore policy privacy (i.e., the content of users’ redaction policies), causing severe privacy leakage threats to users since users’ policies usually contain large amounts of private information (e.g., health conditions and geographical locations) and limiting the applications in IoT systems. To bridge this research gap, we propose PFRB, a policy-hidden fine-grained redactable blockchain in decentralized blockchain-based IoT systems. PFRB follows the decentralized settings and fine-grained chameleon hash-based redaction in existing redactable blockchains. In addition, PFRB hides users’ policies during policy matching such that apart from successful policy matching, users’ policy contents cannot be inferred and valid redactions cannot be executed. Some main technical challenges include determining how to hide policy contents and support policy matching. Inspired by Newton’s interpolation formula-based secret sharing, PFRB converts policy contents into polynomial parameters and utilizes multi-authority attribute-based encryption to further hide these parameters. Theoretical analysis proves the correctness and security against the chosen-plaintext attack. Extensive experiments on the FISCO blockchain platform and IoT devices show that PFRB achieves competitive efficiency over current redactable blockchains.

## 1. Introduction

The Internet of Things (IoT) is defined as connecting all objects through information-sensing devices such as radio frequency identification to the Internet, enabling intelligent recognition and management. The concept of IoT entails integrating sensors into various objects, including power grids and railways, enabling data collection and communication for seamless connectivity and interaction with the physical world [1,2,3]. However, due to the inherent decentralization of IoT device deployment [4,5], achieving secure data management poses challenges.

To address these challenges, blockchain has been widely applied in IoT systems as a decentralized data management platform, providing traceability and integrity to secure IoT systems [6,7,8]. According to a report by Morder Intelligence (https://www.mordorintelligence.com/industry-reports/blockchain-iot (accessed on 31 July 2023)), the market size of blockchain-based IoT systems is expected to grow from USD 568.51 million in 2023 to USD 3436.54 million by 2028. Despite these benefits, immutable blockchains present two limitations in current blockchain-based IoT systems. Firstly, the immutability of blockchain violates the right to be forgotten in the General Data Protection Regulation (GDPR) (https://gdpr-info.eu/art-17-gdpr/ (accessed on 31 July 2023)), severely limiting the development and implementation of blockchain-based IoT systems. GDPR mandates that users should be able to erase their personal data (e.g., health and transportation data) from data management systems. However, with immutable blockchains, once data are appended to the blockchain, no modifications can be made [9]. This conflict between immutability and GDPR could result in high fines for blockchain-based IoT systems. Secondly, IoT devices are vulnerable to network attacks and can be used to spread improper information (e.g., indecent surveillance videos) in blockchain-based IoT systems. If this improper information cannot be redacted, it may negatively impact the blockchain-based IoT application ecology [10,11,12].

To address these limitations, some redactable blockchains have been proposed, adopting chameleon hashes [13,14,15,16,17,18]. Specifically, in 2017, Ateniese et al. [13] introduced the first redactable blockchain by using the chameleon hash technique. Their work extended researchers’ insight into immutable blockchains and made blockchains compliant with data regulation (e.g., GDPR). Unfortunately, their work only supports all-or-nothing redaction privileges, i.e., they either cannot redact any data or can redact all data. Clearly, it is infeasible in practice since real-world blockchain applications contain a large number of devices that own different attributes. To achieve fine-grained redaction, Derler et al. [13], Ma et al. [18], and Xu et al. [19] utilized attribute-based fine-grained access control and chameleon hash to introduce the policy-based chameleon hash technique (PCH), subsequently using PCH to achieve fine-grained redactable blockchains. Following PCH, Mao et al. [18] further introduced a decentralized policy-based chameleon hash technique (DPCH) and used it to design a decentralized fine-grained redactable blockchain. However, in existing fine-grained redactable blockchains, users’ policies are public to all users, making them unsuitable for some policy-sensitive IoT systems, such as IoT-based smart healthcare and smart transportation [20]. In these IoT systems, users’ policies contain sensitive private information, e.g., users’ health conditions and geographical locations [21]. For instance, in an IoT-based smart healthcare application, users use their IoT devices (e.g., smartwatches) to record their health data and utilize blockchain-based IoT systems to manage the data. In the application, users’ policies usually contain information about users’ health conditions, such as sensitive health details [22]. Obviously, if this private information is leaked, users may face discrimination and even potential attacks [23]. Therefore, there is a great need to design decentralized policy-hidden redactable blockchains in blockchain-based IoT systems. We make a comparison between our proposed solution and existing works, as shown in Table 1. We address the issue of policy disclosure in a decentralized, fine-grained setting.

To solve the above problem, we propose a policy-hidden fine-grained redactable blockchain scheme (named PFRB) in decentralized blockchain-based IoT systems. In PFRB, users can enjoy blockchain services while hiding their policies. A technical challenge in designing PFRB is how to hide policy contents and support policy matching. PFRB draws inspiration from Newton’s interpolation formula-based secret sharing and gracefully converts policy contents into polynomial parameters. Particularly, we summarize the main contributions as follows:

- We propose a policy-hidden fine-grained redactable blockchain scheme (named PFRB) for blockchain-based IoT systems. With decentralized settings, PFRB enables users to achieve fine-grained data redaction without compromising policy privacy.
- PFRB leverages multi-authorized attribute-based encryption and Newton’s interpolation formula-based secret sharing to construct a decentralized secret sharing for policy hiding. Then, based on the constructed secret sharing, PFRB further enriches chameleon hashes to achieve decentralized policy-hidden fine-grained redactable blockchains.
- Security analysis proves the security of PFRB under the chosen-plaintext attack in the random oracle model. Experimental results show that PFRB has competitive efficiency over recent fine-grained redactable blockchain schemes.

Organization. The remainder of this paper is structured as follows. In the next section, we review existing works on redactable blockchains to introduce the research gap. In Section 3, we provide an introduction to the preliminary information, followed by an overview of the PFRB system in Section 4. Next, in Section 5, we provide insight into the detailed construction of PFRB. Section 6 presents a formal correctness analysis and security analysis. Performance evaluation of PFRB is shown in Section 7. Finally, concluding remarks are given in Section 8.

## 2. Related Work

This section provides a systematic review of the current literature on redactable blockchain techniques and introduces the research gap.

In 2017, Ateniese et al. [13] introduced the first redactable blockchain by using the chameleon hash technique. Their work extended researchers’ insight into immutable blockchains and made blockchains compliant with the data regulation (e.g., GDPR and CCPA). Unfortunately, their work only supports all-or-nothing redaction privileges, i.e., they either cannot redact any data or can redact all data. Clearly, it is infeasible in practice since real-world blockchain applications contain a large number of devices that own different attributes.

To address the above limitation, Derler et al. [16] proposed a policy-based chameleon hash (PCH) and used PCH to implement fine-grained redactable blockchains. PCH combines chameleon hashes, ephemeral trapdoors, and linear secret sharing matrix-based attribute-based encryption to associate transactions with access policies, enabling rewriting only when editors’ attributes satisfy the policy. Followed by Derler et al.’s work, Tian et al. [24] and Xu et al. [19] extent redactable blockchains with accountability by introducing digital signatures, respectively. Due to the utilization of centralized attribute-based encryption schemes, the above schemes can only be applied in consortium blockchains, and cannot support generally decentralized settings. However, in practical blockchain applications, especially distributed IoT systems, decentralized systems are more general. To achieve decentralized redactable blockchains, Ma et al. [18] introduced the first decentralized policy-based chameleon hash (DPCH) by linear secret sharing matrix-based multi-authority attribute-based encryption and used DPCH to achieve decentralized fine-grained blockchain redaction.

However, redaction policies in the above redactable blockchains are open to all participants, which is infeasible in practical blockchain-based IoT systems since policies in IoT systems usually contain users’ private information. For instance, in IoT-based smart healthcare applications, patients use their smart swatches to collect their health conditions (such as heart rate and personal temperature) and specify policies to allow their private doctors to edit. Evidently, these policies usually contain sensitive information regarding patients’ health conditions and geographical locations. Once this private information is leaked, adversaries can launch attacks on these patients such as robbery and discrimination. Thus, the protection of policy privacy in decentralized redactable blockchains represents an urgent matter.

## 3. Preliminary

In this section, we introduce the building blocks of PFRB, i.e., multi-authority attribute-based encryption, Newton’s interpolation formula-based secret sharing, and chameleon hash.

### 3.1. Multi-Authority Attribute-Based Encryption

A multi-authority attribute-based encryption (MA-ABE) [28] system consists of arbitrary numbers of attribute authorities and users. A set of global public parameters is defined in the system. Users can select an attribute authority and obtain their corresponding decryption key. The authorization authority performs the appropriate attribute key generation algorithm and returns the result to the user. The encryption process uses the global public parameters and a set of attributes to generate the ciphertext. The decryption process uses the decryption key for the attribute set to perform decryption.

Definition 1 (MA-ABE): A multi-authority attribute-based encryption $ABE_{MC}$ involves three types of entities: authorities, data owners, and data users. It includes five algorithms:

- Global Setup (λ)→(GP): This algorithm accepts a secure parameter λ as input and produces a public global parameter GP as output.
- Authority Setup (GP) → (PK, SK): In this step, the algorithm takes the public global parameter GP as input and generates a public key PK and a secret key SK as output. It is crucial to keep the secret key SK confidential, while the public key PK is intended for publication.
- Encryption (M,(A,ρ),GP,{PK})→(CT): The algorithm accepts several inputs, including a message *M*, an n×ℓ access matrix *A* with ρ mapping its rows to attributes, the global parameter GP, and the public keys of the relevant authorities PK. It then produces a ciphertext CT as output.
- KeyGen (ID, *i*, SK, GP) $\to (K_{i,ID}$): The algorithm generates a key $K_{i,ID}$ for attribute *i* associated with an authority using the inputs: a global identifier ID, the attribute *i*, the secret key SK, and the public global parameter GP.
- Decryption ($CT,\{K_{i,ID}\},GP$) →(M): The algorithm decrypts the ciphertext CT using the input parameters: the key $K_{i,ID}$ for ID and attribute *i*, as well as the global parameter GP. The result of the decryption process is the message *M*.

### 3.2. Newton’s Interpolation Formula-Based Secret Sharing

In this paper, Newton’s interpolation formula is primarily used for key recovery. Also, due to the introduction of polynomial-oriented secret sharing, PFRB achieves higher efficiency than traditional linear secret sharing matrix-based works. The transaction issuer hides the key within the zeroth term of a polynomial, ensuring that only users who meet the policy requirements can reconstruct the polynomial and access the hidden key.

- Secret Generation: Assume that there are (n+1) points represented as $\left(x_{0},y_{0}\right),\left(x_{1},y_{1}\right),$$\ldots ,(x_{n},y_{n})$. Here, $x_{i}$ is called the interpolation point, and $y_{i}$ is called the interpolation value. Given an interpolation polynomial f(x), for each i=0,1,2,…,n, $y_{i}$ is represented as $y_{i}=f\left(x_{i}\right)$. The Newton’s basis $n_{i}\left(x\right)$ is defined as follows:
  $$n_{i}\left(x\right)=\left\{\begin{array}{cc} 1, & \mathrm{if}\;i=0,\\ \prod_{j=0}^{i-1}\left(x-x_{j}\right), & \mathrm{otherwise}. \end{array}\right.$$
  Based on $n_{i}\left(x\right)$, the Newton’s interpolation polynomial $Q_{n}\left(x\right)$ can be defined as follows:
  $$Q_{n}\left(x\right)=K_{0}+K_{1}\left(x-x_{0}\right)+\cdots +K_{n}\prod_{i=0}^{n-1}\left(x-x_{i}\right)=\sum_{i=0}^{n}K_{i}n_{i}\left(x\right).$$
  Specifically, based on $x_{0}$, $Q_{n}\left(x\right)$ can be estimated as follows:
  $$Q_{n}\left(x_{0}\right)=\sum_{i=0}^{n}K_{i}n_{i}\left(x\right)=K_{0}=f\left(x_{0}\right)=f\left[x_{0}\right].$$
  where $f[x_{0}]$ represents the zeroth order divided difference. Similarly, based on $x_{1}$, the Newton’s interpolation polynomial $Q_{n}\left(x\right)$ is estimated as follows:
  $$Q_{n}\left(x_{1}\right)=K_{1}\left(x-x_{0}\right)+K_{0}=K_{1}\left(x-x_{0}\right)+f\left[x_{0}\right]=f\left[x_{1}\right].$$
  Thus, $K_{1}$ can be estimated as follows:
  $$K_{1}=f\left[x_{0},x_{1}\right]=\frac{f\left[x_{1}\right]-f\left[x_{0}\right]}{x_{1}-x_{0}}.$$
  where $f[x_{0},x_{1}]$ represents the first order divided difference. Without loss of generality, $K_{i}$ can be defined as follows:
  $$K_{i}=\frac{f\left[x_{1},x_{2},\ldots ,x_{i}\right]-f\left[x_{0},x_{1},\ldots ,x_{i-1}\right]}{x_{i}-x_{0}}.$$
  where $f[x_{1},x_{2},\ldots ,x_{i}]$ denotes the *i*-th order divided difference, respectively. For more details, the reader can refer to previous literature.
- Secret Construction: We can reconstruct the secret with Newton’s parameters as follows:
  $$s\;=\;Q_{n}\left(0\right)\;=\;\sum_{i=0}^{n}K_{i}n_{i}\left(0\right).$$

### 3.3. Chameleon Hash

A chameleon hash CH typically encompasses the following five algorithms:

- $\mathrm{Setup}(1^{\lambda })\to pp$: The probabilistic setup algorithm takes a security parameter λ as input and generates a public parameter pp as output. The public parameter pp is used in subsequent algorithms and protocols to ensure the security and functionality of the system.
- KeyGen(pp)→(pk,sk): The probabilistic key generation algorithm takes the public parameter pp as input and generates a public-secret key pair (pk,sk) as output. The public key pk is used for encryption or other public operations, while the secret key sk is kept confidential and used for decryption or other sensitive operations.
- Hash(pk,m)→(h,r): The probabilistic hash algorithm takes the public key pk and a message m∈M as input. It then produces an output of 1 if the tuple (h,r) is considered valid according to the algorithm’s criteria. If the tuple is not valid, the output will be 0.
- Verify(pk,m,h,r)→{0,1}: The deterministic verification algorithm takes the public key pk, message *m*, hash value *h*, and randomness value *r* as input. It then determines whether the tuple (h,r) is valid according to the defined criteria. If the tuple is valid, the algorithm outputs 1. Otherwise, if the tuple is not valid, the output will be 0.
- $\mathrm{Adapt}\left(sk,m,m^{\prime },h,r\right)\to r^{\prime }$: The deterministic adaptation algorithm takes the secret key sk, message m∈M, hash value *h*, and randomness value *r* as input. It then generates an adapted randomness value $r^{\prime }$ as output.

## 4. System Overview

In this section, we present the system model, brief definition, and security model of PFRB.

### 4.1. System Model

**Figure 1:**
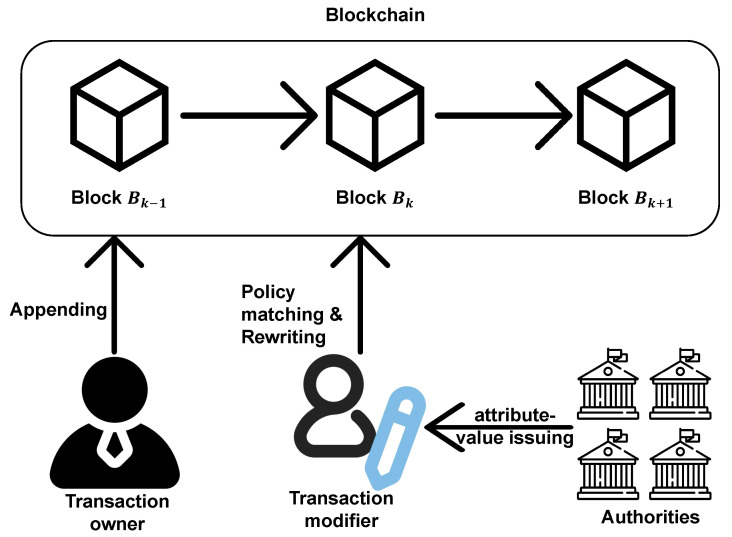
PFRB system model.

As shown in Figure 1, the PFRB (Privacy-Preserving Redactable Blockchain) system involves four types of entities:

- Authorities: The authorities are all trusted. One of them initializes the system, and they can all generate attribute-value pairs.
- Transaction Owner: The transaction owner is also trusted and wants to place a deal or some data on the blockchain. They hash the data and attempt to add the transaction to the blockchain.
- Transaction Modifier: The transaction modifier is a user who wants to modify a transaction in the blockchain. They retrieve the attribute-value pairs and try to match the transaction to modify it.
- Blockchain Participants: The blockchain participants are users of the redactable blockchain. They verify each transaction published by the transaction owners or the transaction modifiers.

### 4.2. Definition of PFRB

We next provide a brief definition of PFRB and summarize the notations in Table 2.

- Setup ($1^{\lambda }$)→($pp_{s}$, $pk_{s}$, $sk_{s}$): Given a security parameter, the Setup algorithm outputs a public parameter $pp_{s}$, public key $pk_{s}$, and secret key $sk_{s}$. Then, the authority publishes $pp_{s}$ and $pk_{s}$ to all users.
- RKGen (msk,ρ)→($dk_{\rho },\left\{\Delta_{i,R_{j}}\right\}$): The RKGen algorithm takes msk and ρ as input, where ρ is the attribute set of the modifier. The algorithm outputs the decryption key $dk_{\rho }$ and the Lagrange coefficients of ρ, $\left\{\Delta_{i,R_{j}}\right\}$.
- ModSetup ($sk_{s},id$)→($sk_{id},\sigma_{id}$): The ModSetup algorithm takes the secret key $sk_{s}$ and the global identifier id as input. It outputs the modifier’s secret key $sk_{id}$ and the modifier’s signature $\sigma_{id}$.
- AuthSetup (θ)→($pk_{\theta },sk_{\theta }$): The AuthSetup algorithm takes the authority θ as input and outputs the authority’s public key $pk_{\theta }$ and secret key $sk_{\theta }$.
- ModKeyGen ($pk_{s},id,\sigma_{id},sk_{\theta },A$)→$sk_{id,A}/⊥$: The ModKeyGen algorithm takes the public key $pk_{s}$, the modifier’s global identifier id, the modifier’s signature $\sigma_{id}$, the authority’s secret key $sk_{\theta }$, and an attribute *A* as input. It generates the secret key $sk_{id,A}$ for the modifier’s attribute *A* if the request is legal; otherwise, it outputs nothing.
- Hash ($pk_{s},\left\{pk_{\theta }\right\},m,R$)→($pk^{etd},h,r,c$): The Hash algorithm is designed to take the following inputs: the public key $pk_{s}$, a group of authorities’ public keys $pk_{\theta }$, the message *m* to be encrypted, and the policy *R* of the target receiver. It generates four outputs: a public key $pk^{etd}$ (a public component of the ephemeral trapdoor), a hash value *h*, a randomness value *r*, and a ciphertext *c*. The ciphertext *c* plays the crucial role of securely sealing the secret component $sk^{etd}$, guaranteeing its confidentiality.
- Verify ($pk_{s},pk^{etd},m,h,r$)→{0,1}: The Verify algorithm can be executed by any entity within the system. It accepts the following inputs: the public key $pk_{s}$, the public component $pk^{etd}$ of the ephemeral trapdoor, the message m∈M, the hash value *h*, and the randomness value *r*. The algorithm then determines whether the tuple (h,r) is valid according to its defined criteria. If the tuple is deemed valid, the algorithm outputs 1. However, if the tuple is found to be invalid, the output will be 0.
- Adapt ($sk_{id},\left\{sk_{id,A}\right\},c,m,m^{\prime },h,r$)→r’: The Adapt algorithm is executed by the transaction modifier. It takes inputs such as the secret component $sk^{etd}$, a set of secret keys $sk_{id,A}$, the ciphertext *c*, messages *m* and $m^{\prime }$, the hash value *h*, and the randomness value *r*. The output of the algorithm is a new randomness value $r^{\prime }$.

### 4.3. Security Model

In our scheme, we assume that all authorities and the data owner are trusted entities, and communications between them are secure. The transaction owner generates mutable transactions honestly, and the authorities preserve the secret key honestly. However, other entities, such as chain participants, can act as adversaries and collaborate to launch the chosen-plaintext attack. The security of PFRB is defined as the indistinguishability and the collision resistance under the chosen-plaintext attack in the random oracle model as follows.

- Setup: The challenger runs the Setup algorithm and shares the public parameters PK with the adversary.
- Phase 1: The challenger allows the adversary to request private keys from the encryption oracle $O_{E}$ by their attributes $S_{1},\ldots ,S_{q1}$.
- Challenge: The adversary selects and uploads two messages, $M_{0}$ and $M_{1}$, of equal length. The adversary also presents a challenge access structure, denoted as *A*, which none of the previously generated attribute sets can satisfy. The challenger randomly chooses a coin flip outcome, encrypts either $M_{0}$ or $M_{1}$ under the challenge access structure *A*, and provides the resulting ciphertext $CT^{*}$ to the adversary.
- Phase 2: Phase 1 is repeated, but with the additional constraint that none of the sets of attributes $S_{q1+1},S_{q1+2},\ldots ,S_{q}$ satisfy the access structure associated with the given challenge. This restriction ensures that the adversary cannot find any new sets of attributes that fulfill the challenge access structure.
- Guess: Based on the above experiment, the adversary outputs a guess, $b_{0}$, of *b*.

We say that the adversary A wins the above game if the guess $b^{\prime }$ equals *b*. Specifically, PFRB is secure against the chosen-plaintext attack (CPA) if any probabilistic polynomial-time adversary A only has a negligible advantage to win the game as follows.

$$\begin{array}{c} {\mathrm{Adv}}_{A}^{CPA}\left(\lambda \right)=\left|\mathrm{Pr}\left[b^{\prime }=b|A^{O_{E}}\left(M_{0},M_{1},CT^{*}\right)\right]\right|. \end{array}$$

## 5. Proposed Scheme

**Figure 2:**
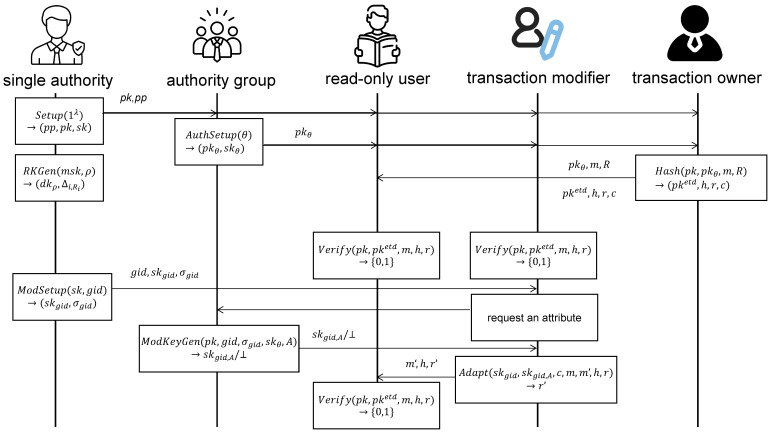
Detailed workflow in PFRB.

In this section, we present detailed construction of PFRB. The workflow of PFRB is shown in Figure 2. There are eight algorithms: Setup, RkGen, ModSetup, AuthSetup, ModKeyGen, Hash, Verify and Adapt.

- Setup ($1^{\lambda }$)→($pp_{s},pk_{s},sk_{s},mpk,msk$):
  - Given a security parameter λ, generate the bilinear group description ($p,G,G_{0},e,g$).
  - Running $RSAKeyGen(1^{\lambda })$, and then get the first set of RSA parameter ($n_{0}$, $p_{0}$, $q_{0}$, $e_{0}$, $d_{0}$)
  - Choose three random exponents $\alpha \in Z_{p},\beta ,\gamma \in G$, a mapping function, and four hash functions as follows.
    $$\begin{array}{cc} \; & H_{b,Z_{p}}:\left\{0,1\right\}\to Z_{p},\\ \; & H_{ID,G}:\left\{0,1\right\}\times ID\to G,\\ \; & H_{0}:{\left\{0,1\right\}}^{*}\to Z_{n}^{*},\\ \; & H_{U,G}:U\to G,\\ \; & F_{m}:U\to U_{\theta }. \end{array}$$
  - Set $Y_{\beta }=e{\left(g,g\right)}^{\beta },Y_{\gamma }=e{\left(g,g\right)}^{\gamma }$. Choose *n*, *d* and 2n+2 random values $t_{1},\cdots ,t_{n+1}$, $d_{1},\cdots ,d_{n+1}\in Z_{p}$ and set $T_{i}=g^{t_{i}},D_{i}=g^{d_{i}}$ for each *i* from 1 to n+1.
  - Choose a symmetric encoding method $F_{en}:{\left\{0,1\right\}}^{*}\to G_{0}$, and the corresponding decoding method $F_{en}^{-1}:G_{0}\to {\left\{0,1\right\}}^{*}$.
  - Calculate the system public parameter $pp_{s}$, master secret key msk, master public key mpk, system public key $pk_{s}$, and system secret key $sk_{s}$ as:
    $$\begin{array}{cc} \; & pp_{s}=\left(1^{\lambda },p,G,G_{0},e,g,H_{b,Z_{p}},H_{ID,G},H_{0},H_{U,G},F_{m},F_{en},F_{en}^{-1}\right),\\ \; & mpk=\left(Y_{\beta },Y_{\gamma },\left\{T_{i}\right\},\left\{D_{i}\right\}\right),msk=\left(\beta ,\gamma \right),\\ \; & pk_{s}=\left(H_{0},n_{0},e_{0},g^{\alpha }\right),sk_{s}=\left(d_{0},\alpha \right). \end{array}$$
    The function $H_{ID,G}$ receives a bit b∈{0,1} and an input id∈ID, producing a hash value in *G*. In our system, the attributes are named using the format “[attribute-id]@[authority-id]”. To extract only the authority ID from the attribute name, we use a mapping function called $F_{m}$. This function helps us retrieve the authority ID while ignoring the attribute ID.
- RKGen $\left(msk,\rho \right)\to (dk_{\rho },\left\{\Delta_{i,R_{j}}\right\})$: ρ is an attribute set of modifier.
  - Randomly generate a d-1 degree polynomial $q_{2}$ with $q_{2}\left(0\right)=\gamma$
  - Calculate the values of $dk_{i}=g^{\frac{q_{2}\left(i\right)}{d_{i}}}$ for each *i* in ρ, and store them as $dk_{\rho }=\left\{dk_{i}\right\}$.
  - Compute a set of Lagrange coefficients $\Delta_{i,R_{j}}$, where each Lagrange coefficient might satisfies the policy, and $R_{j}$ belongs to ρ.
- ModSetup $\left(sk_{s},id\right)\to \left(sk_{id},\sigma_{id}\right)$:
  - The authorities compute their secret key $sk_{id}=d_{0}$ and their signature $\sigma_{id}=H_{ID,G}{\left(1,id\right)}^{\alpha }$. Return $sk_{id}$ and $\sigma_{id}$.
- AuthSetup $\left(\theta \right)\to \left(pk_{\theta },sk_{\theta }\right)$:
  - The authority Chooses two random values $a_{\theta }$, $b_{\theta }\in Z_{p}$. Calculate the secret key $sk_{\theta }$ as $\left(a_{\theta },b_{\theta }\right)$ and public key $pk_{\theta }$ as $\left(e{\left(g,g\right)}^{a_{\theta }},g^{b_{\theta }}\right)$. Return $pk_{\theta }$ and $sk_{\theta }$.
- ModKeyGen $\left(pk_{s},id,\sigma_{id},sk_{\theta },A\right)\to sk_{id,A}/⊥$
  - If $e\left(g,\sigma \right)\neq e(g^{\alpha },H_{ID,G}\left(1,id\right))$, return ⊥.
  - Generate a random value $t\in Z_{p}$. Compute $sk_{id,A,0}=g^{a_{\theta }}H_{ID,G}{\left(0,id\right)}^{b_{\theta }}H_{U,G}{\left(A\right)}^{t}$ and $sk_{id,A,1}=g^{t}$.
  - Output the corresponding secret key of the attribute *A* as follows.
    $$sk_{id,A}=\left(id,A,sk_{id,A,0},sk_{id,A,1}\right).$$
- $Hash\left(pk_{s},\left\{pk_{\theta }\right\},m,R\right)\to \left(pk^{etd},h,r,c\right)$: R is a policy of target receiver.
  - Run the RSA key generator RSAKeyGen($1^{\lambda }$) and generate another set of RSA parameter $\left(n_{1},p_{1},q_{1},e_{1},d_{1}\right)$.
  - Choose $H_{1}:{\left\{0,1\right\}}^{*}\to Z_{n_{1}}^{*},r_{0}\in Z_{n_{0}}^{*}$ and $r_{1}\in Z_{n_{1}}^{*}$. Then compute two hash values $h_{0}=H_{0}\left(m\right)r_{0}^{e_{0}}$ and $h_{1}=H_{1}\left(m\right)r_{1}^{e_{1}}$
  - Choose a random sequence $r_{t}\in {\left\{0,1\right\}}^{\lambda }$. Then, run $SE.KeyGen(1^{\lambda })$ to generate a key *k*. Subsequently, utilize *k* to generate a ciphertext $c_{t}\leftarrow SE.Enc\left(k,d_{1}\right)$.
  - Run the symmetric encryption algorithm and compute $k_{c}\leftarrow F_{en}\left(k,r_{t}\right)$ and $z\leftarrow H_{b,Z_{p}}\left(r_{t},G_{n}\left(x\right)\right)$. Calculate $c_{0}=k_{c}e{\left(g,g\right)}^{z}$. Choose a set of random numbers $u_{r},t_{r},r_{1,r},r_{2,r}\in Z_{p}$. Compute
    $$T_{r}=g^{t_{r}},U_{r}=g^{u_{r}},R_{1,r}=g^{r_{1,r}},R_{2,r}=g^{r_{2,r}}.$$
    Compute $P_{i,r}=D_{i,r}^{r_{1,r}}$, $E_{i,r}=T_{i,r}^{r_{2,r}}$ where i∈R.
  - Compute
    $$\begin{array}{cc} \; & K_{1,r}=e\left(R_{1,r},T_{r}\right)Y_{\gamma }^{r_{1,r}},\\ \; & K_{2,r}=e\left(R_{2,r},U_{r}\right)Y_{\beta }^{r_{2,r}},\\ \; & V_{r}=z⊕H\left(e\left(R_{1,r},T_{r}\right)\right)⊕H\left(e\left(R_{2,r},U_{r}\right)\right). \end{array}$$
  - Return the public key $pk^{etd}$, random value *r*, hash value *h*, and ciphertext *c* as follows.
    $$\begin{array}{cc} \; & pk^{etd}=\left(H_{1},n_{1},e_{1}\right),h=\left(h_{0},h_{1}\right),r=\left(r_{0},r_{1}\right),\\ \; & c=(c_{0},T_{r},U_{r},\left\{P_{i,r}\right\},\left\{E_{i,r}\right\},W_{r},\left\{K_{1,r}\right\},\left\{K_{2,r}\right\},V_{r}). \end{array}$$
- Verify $\left(pk_{s},pk^{etd},m,h,r\right)\to \left\{0,1\right\}$:Parse $h=(h_{0},h_{1})$ and $r=(r_{0},r_{1})$
  - Return 1 if $h_{0}=H_{0}\left(m\right)r_{0}^{e_{0}}\;mod\;n_{0}$ and $h_{1}=H_{1}\left(m\right)r_{1}^{e_{1}}\;mod\;n_{1}$; otherwise, return 0;
- Adapt $\left(sk_{id},\left\{sk_{id,A}\right\},c,m,m^{\prime },h,r\right)\to r^{\prime }$:
  - If *m* equals $m^{\prime }$, then output $r^{\prime }\;=\;r$.
  - Enumerate all sets of combinations of attributes and compute
    $$\begin{array}{cc} \; & g_{T,1}=K_{1}/\prod_{i,R_{j}}e{\left(P_{i},dk_{i}\right)}^{\Delta_{i,R_{j}}\left(0\right)},with\;R_{j}\subset R,\left|R_{j}\right|\geq d,\\ \; & g_{T,2}=K_{2}/e\left(\prod_{i,S}E_{i,r},W_{r}\right),\\ \; & z=V⊕H\left(g_{T,1}\right)⊕H\left(g_{T,2}\right),\\ \; & k_{c}=c_{0}/e{\left(g,g\right)}^{z},\left(k,r_{t}\right)\leftarrow F_{en}^{-1}\left(k_{c}\right). \end{array}$$
  - Run $d_{1}\leftarrow SE.Dec\left(c_{t},k\right)$. Compute
    $$\begin{array}{cc} \; & r_{0}^{\prime }={\left(h_{0}\left(H_{0}{\left(m^{\prime }\right)}^{-1}\right)\right)}^{d_{0}}mod\;n_{0},\\ \; & r_{1}^{\prime }={\left(h_{1}\left(H_{1}{\left(m^{\prime }\right)}^{-1}\right)\right)}^{d_{1}}mod\;n_{1}. \end{array}$$
  - Return the randomness $r^{\prime }=\left(r_{0}^{\prime },r_{1}^{\prime }\right)$.

## 6. Theoretical Analysis

In this section, we theoretically analyze the correctness and security of PFRB. Then, we discuss some promising applications of PFRB.

### 6.1. Correctness Analysis

In this section, we will provide detailed proof of the correctness of the proposed scheme in this paper. The scheme presented in this paper builds upon Ma et al’s scheme (i.e., RBDS22) [18] while incorporating additional improvements. It is worth noting that if RBDS22 is correct and the calculations of $K_{1,r}$ and $K_{2,r}$ in this scheme are correct, then the proposed scheme in this paper is also correct. We will now present the specific proof of the correctness of $K_{1,r}$ and $K_{2,r}$ as follows:

In the Hash part, we have $K_{1,r}=e\left(R_{1,r},T_{r}\right)Y_{\gamma }^{r_{1,r}}$, $K_{2,r}=e\left(R_{2,r},U_{r}\right)Y_{\beta }^{r_{2,r}}$. $K_{1,r}$ can be transformed as follows:

$$\begin{array}{c} K_{1,r}=e\left(R_{1,r},T_{r}\right)Y_{\gamma }^{r_{1,r}}=e\left(R_{1,r},T_{r}\right)e{\left(g,g\right)}^{\gamma ,r_{1,r}}. \end{array}$$

Similarly, $K_{2,r}$ can be transformed as follows:

$$\begin{array}{c} K_{2,r}=e\left(R_{2,r},U_{r}\right)Y_{\beta }^{r_{2,r}}=e\left(R_{2,r},U_{r}\right)e{\left(g,g\right)}^{\beta ,r_{2,r}}. \end{array}$$

In the Adapt part, we have: $g_{T,1}=K_{1,r}/\prod_{i,R_{j}}e{\left(P_{i,r},dk_{i}\right)}^{\Delta i,R_{j}\left(0\right)}$, $g_{T,2}=K_{2,r}/$$e\left(\prod_{i,s}E_{i,r},W_{r}\right)$.

Transform these two parts as follows:

$$\begin{array}{cc} \; & K_{1,r}=g_{T,1}\cdot \prod_{i,R_{j}}e{\left(P_{i,r},dki\right)}^{\Delta i,R_{j}\left(0\right)}=g_{T,1}\cdot \prod_{i,R_{j}}e\left(P_{i,r},dki^{\Delta i,R_{j}\left(0\right)}\right)\\ \; & =g_{T,1}\cdot \prod_{i,R_{j}}e\left(D_{i,r}^{r_{1,r}},dki^{\Delta i,R_{j}\left(0\right)}\right)=g_{T,1}\cdot \prod_{i,R_{j}}e\left(g^{d_{i,r}},dki^{\Delta i,R_{j}\left(0\right)}\right)\\ \; & =g_{T,1}\cdot \prod_{i,R_{j}}e\left(g^{d_{i,r}},g^{\frac{q_{2}\left(i\right)}{d_{i}}\Delta_{i,R_{j}}\left(0\right)}\right)=g_{T,1}\cdot e{\left(g,g\right)}^{\gamma ,r_{1,r}}. \end{array}$$

Similarly, we have $K_{2,r}=g_{T,2}\cdot e{\left(g,g\right)}^{\beta ,r_{2,r}}$.

When assuming that the two instances of $K_{1,r}$ and $K_{2,r}$ are equal, we can deduce that $e\left(R_{1,r},T_{r}\right)=g_{T,1}$ and $e\left(R_{2,r},U_{r}\right)=g_{T,2}$. In this case, the modifier can obtain the desired decryption key *z* from *V* as follows: $z=V⊕H\left(g_{T,1}\right)⊕H\left(g_{T,2}\right)$.

From the above, it is evident that when the calculations of $K_{1,r}$ and $K_{2,r}$ are correct, the modifier can successfully recover the key *z*, thereby reducing the correctness of this paper’s scheme to the correctness of RBDS22. As RBDS22 is known to be correct, it follows that this paper’s scheme is also correct.

### 6.2. Security Analysis

**Theorem** **1.** *PFRB is secure against the chosen-plaintext attack.*

**Proof.**  To prove the security of the encryption method against the chosen-plaintext attack, we need to demonstrate that the adversary cannot distinguish the generated ciphertext, even if it can choose a set of plaintexts and observe their encryption forms.

Considering the message need to be encrypted *m*, the challenger first generates a randomness $r_{t}$. Calculates the $K_{c}$ by running $F_{en}\left(m,r_{t}\right)$. then let *z* = $H_{b,Z_{p}}\left(r_{t},G_{n}\left(x\right)\right)$, where $G_{n}\left(x\right)$ is an invariant polynomial which A cannot get. Computing $c_{0}$ = $k_{c}e{\left(g,g\right)}^{z}$. Return $c_{0}$ to the adversary A.

Recall the above encryption process, we know that e(), *g*, $F_{en}$, $H_{b,Z_{p}}\left(\right)$, λ are fixed. message *m* are values chosen by A, and $r_{t}$ are one-time random value chosen by C. It is obvious that $C_{0}$ is a random value associated with $r_{t}$, which means although the adversary A can continuously submit requests to C and receive their corresponding ciphertexts, the ciphertexts appear random to A. Therefore, A is incapable of distinguishing plaintexts from ciphertexts and it can only randomly guess $b^{\prime }$ from 0,1. Hence, we can know that

$$\begin{array}{c} |{\mathrm{Adv}}_{A}\left[b^{\prime }=b\right]-\frac{1}{2}|\leq \epsilon , \end{array}$$

where ϵ is negligible. Thus, PFRB is of CPA security, and Theorem 1 is proven. □

Also, PFRB can resist user collusion attacks if the soundness of Newton’s interpolation formula-based secret sharing is hard. As shown in the detailed construction, PFRB relies on Newton’s interpolation formula-based secret sharing to achieve controllable redaction. Specifically, Newton’s interpolation formula-based secret sharing is used to specify policies and generate secret keys. namely, the user collusion resistance of PFRB can be reduced to the soundness of Newton’s interpolation formula-based secret sharing. As proven in prior works, Newton’s interpolation formula-based secret sharing holds soundness. Thus, PFRB is secure against the user collusion attack.

### 6.3. Application Discussion

The emergence of decentralized policy-hidden fine-grained redactable blockchain (PFRB) technology has opened new avenues for secure data management and privacy preservation. In this section, we explore the potential benefits of integrating PFRB with smart healthcare, smart industry, and artificial intelligence (AI) systems.

#### 6.3.1. Application of PFRB in Smart Healthcare

Currently, with policy privacy, PFRB can be widely applied in smart healthcare applications to collect and manage medical data. For instance, PFRB ensures that nobody can infer users’ private information from users’ redaction policies. This benefit can impel users’ enthusiasm for using portable devices (e.g., mobile phones and smart swatches) to collect data associated with their health condition, such as heart rate and temperature [29]. Clearly, these data hold high value for medical data analytic applications.

#### 6.3.2. Application of PFRB in Smart Industry

In the industry environment, due to the harsh environment and heavy data collection tasks, IoT devices have been deployed to replace humans’ work and securely transmit their data through blockchains [30]. In this case, policies from IoT devices usually contain large amounts of commercially sensitive data, such as data types and factory addresses [31]. Protecting this information is crucial to increase the wide implementation of blockchain-based IoT systems in the smart industry. Matching these practical requirements, PFRB is a promising solution to blockchain-based IoT systems in the smart industry.

#### 6.3.3. Application of PFRB in AI

Recently, some cases have been proposed to prove the practical feasibility of combining the advantages of blockchains, AI, and IoT systems, such as swarm learning and machine/deep-learning-based IoT systems [32]. Specifically, IoT systems are responsible for collecting data for AI models, and blockchains are responsible for managing data and training AI models. The trained models can then provide rich services for IoT devices, such as fault detection and inference [33]. Similarly, in these promising applications, redaction policies from IoT devices contain much private information of IoT owners, such as policies for personal temperatures in medical analytics containing users’ health conditions. PRFB addresses the policy leakage problem and can further impel the development of combining blockchains, IoT systems, and AI in real-world applications.

## 7. Performance Evaluation

In this section, we conduct experiments on the FISCO blockchain platform to evaluate the practical efficiency of PFRB.

### 7.1. Experimental Settings

Configuration: The experiment was conducted on a personal computer running Windows 10 (x64) with an Intel i7 8550U processor clocked at 1.80GHz and 8GB of memory. The implementation was done using the JPBC library in Java 8, and the MNT224 curve, known for its type-III properties and offering a 96-bit security level, was selected for pairing operations. Additionally, a 2048-bit RSA group was used for the Chameleon hash, providing a security level of 112 bits. Our scheme was deployed on the FISCO BCOS platform, which is an open-source platform customized for the financial industry. It is built upon the BCOS platform and incorporates module upgrades and functionality customization. The “Arbitration Chain” in FISCO leverages blockchain decentralization, tamper resistance, and trustworthiness. Real-time preserved data are securely stored on the blockchain using distributed data storage and encryption algorithms, ensuring the authenticity, legality, and relevance of the evidence.

Parameter design: The data owner randomly selects a news headline and uploads it to the service node as a transaction. In our experiment, we assume that each user can choose up to 100 attributes, such as gender, age, region, education level, occupation, etc., for access permissions. The access control policies for transactions are determined based on information such as the time and location of the news, and up to 100 policies can be set. It is important to note that the traditional scheme lacks policy protection and is vulnerable to security issues such as privacy leaks. This paper presents the experimental performance of key generation, hashing, and adaptation and chooses recent related works [16,18] as comparisons.

Dataset: The implementation of our proposed solution utilizes the MNH9 dataset, which is derived from a real-world open dataset provided by the Australian Broadcasting Corporation. The MNH9 dataset consists of millions of news headlines.

### 7.2. Experimental Results

In this paper, we present a superior solution that excels in key generation, hashing, verification, and adaptation compared to traditional methods.

**Figure 3:**
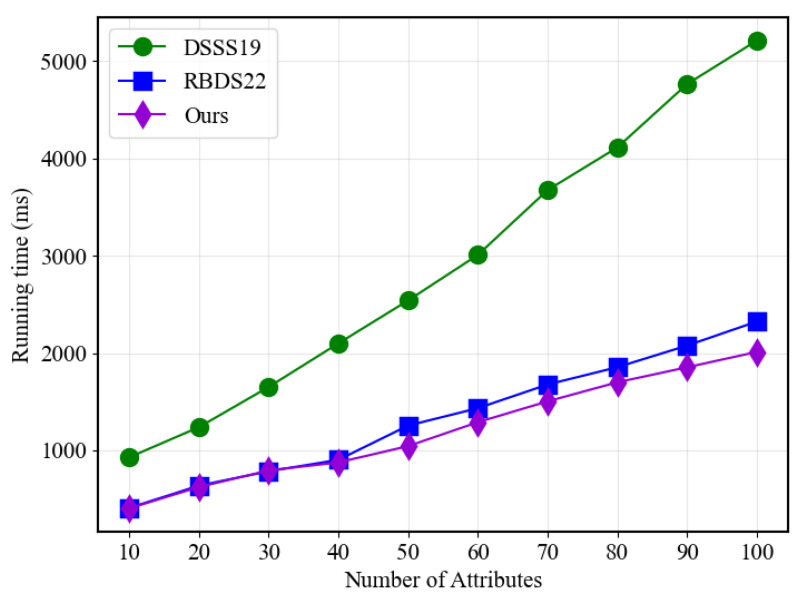
Key generation performance.

Figure 3 illustrates a significant improvement in the key generation aspect. For all three approaches, the required time increases linearly with the number of attributes, and both our proposed scheme and RBDS22 outperform the traditional approach by requiring considerably less time. This noteworthy enhancement is primarily attributed to the higher efficiency of our scheme, which leverages traditional linear secret sharing matrices at the underlying level, resulting in a more stable and efficient process.

**Figure 4:**
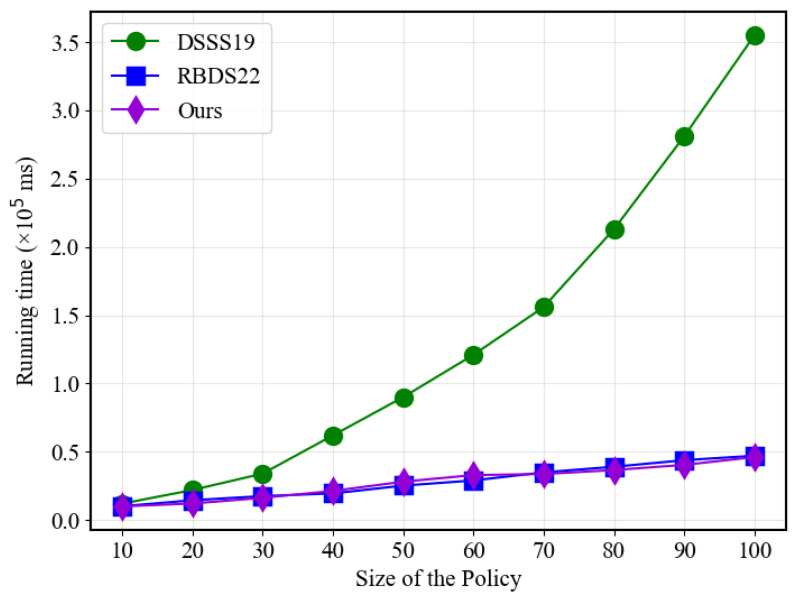
Hash performance with different policy sizes.

Moving on to Figure 4, the most noticeable efficiency improvement of the proposed solution is in the hashing part when compared to the traditional scheme. The traditional approach’s required time increases exponentially with the number of policies, while the proposed solution and RBDS22 demonstrate linear growth. This advantage can be attributed to the use of polynomial functions generated from point values, which have proven to be significantly more efficient than using traditional linear secret sharing matrices schemes.

**Figure 5:**
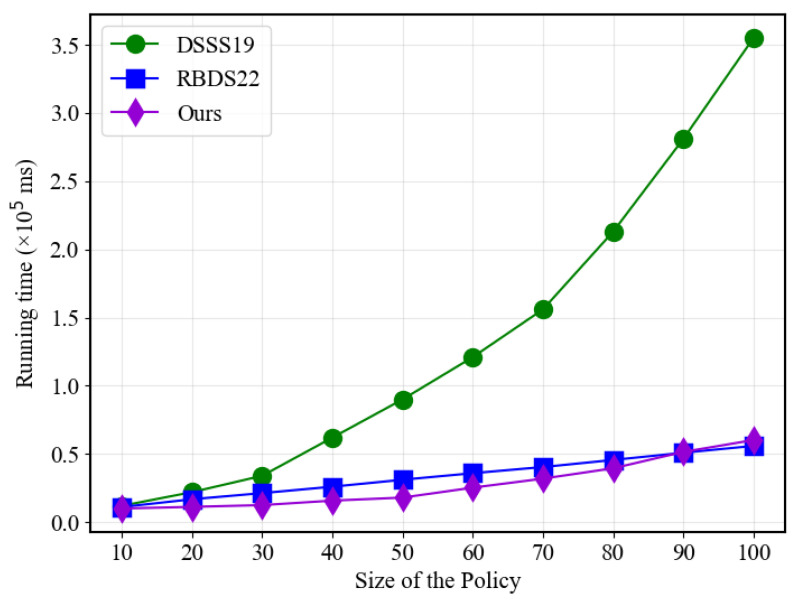
Adaption performance with different policy sizes.

Figure 5 reveals that the adaptation algorithm part, which employs Newton’s interpolation to reconstruct polynomials, presents challenges due to the enumeration of attribute-value pairs owned by the user. While the required time for both the traditional scheme and the proposed solution increases exponentially with the number of policies, the proposed scheme still outperforms the traditional approach significantly. Moreover, RBDS22 shows linear growth in the required time, and the proposed solution performs better than RBDS22, especially when the number of policies is low.

**Figure 6:**
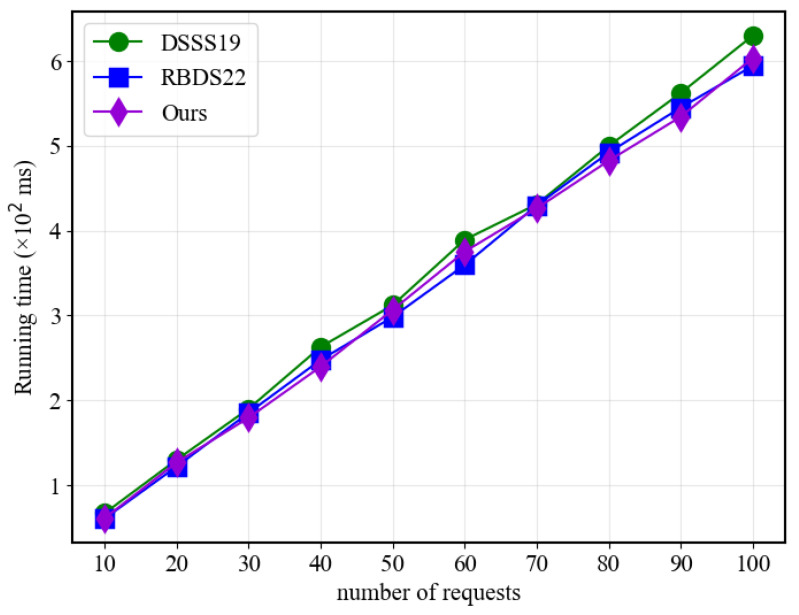
Verify performance with different request numbers.

Figure 6 demonstrates that in the Verify algorithm part, the response time of all three schemes increases linearly with the number of requests, with no significant difference in the time used. This similarity is mainly due to the similarity in the calculation operations performed in the Verify algorithm part of all three schemes. Therefore, the time used by all three schemes is similar in this regard.

**Figure 7:**
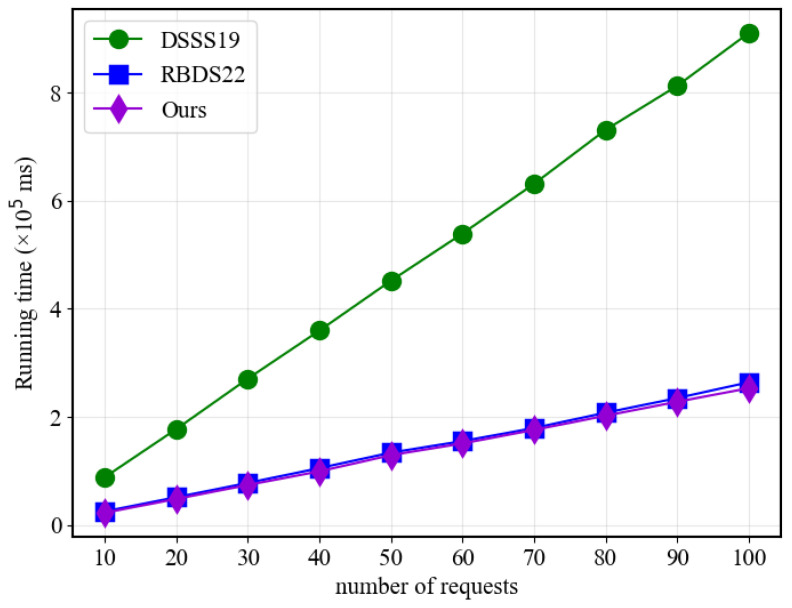
Hash performance with different user numbers.

**Figure 8:**
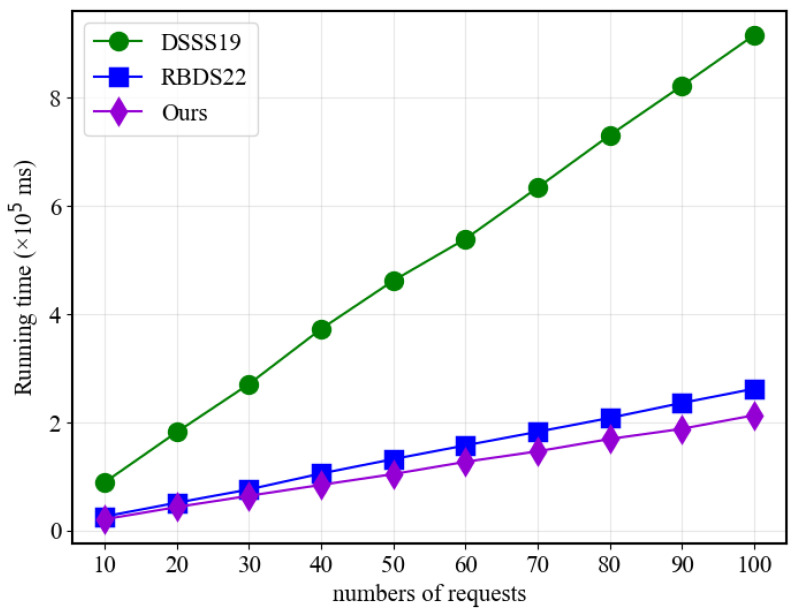
Adaption performance with different user numbers.

Figure 7 and Figure 8 display that when the number of policies is fixed, the response time of all three schemes in the hashing and adaptation algorithm parts increases linearly with the number of requests. However, the proposed scheme requires significantly less time than the traditional scheme and slightly less time than RBDS22 when the number of policies is relatively small. This advantage can be attributed to the proposed scheme’s utilization of MA-ABE and Newton’s interpolation, which enables it to achieve higher efficiency compared to traditional ABE and linear secret sharing matrices.

## 8. Conclusions

In this paper, we propose a policy-hidden fine-grained redactable blockchain (named PFRB) in decentralized blockchain-based IoT systems. Considering existing redactable blockchains, PFRB supports decentralized settings and fine-grained chameleon hash-based redaction. In addition, PFRB ensures that apart from successful policy matching, anyone cannot infer users’ policy contents and execute any valid redaction. PFRB draws inspiration from Newton’s interpolation formula-based secret sharing to convert policy contents into polynomial parameters. PFRB then utilizes multi-authority attribute-based encryption to hide these parameters further. Theoretical analysis proves that PRFB is secure against the chosen-plaintext attack. Extensive experiments on the FISCO blockchain platform and IoT devices show that PFRB achieves competitive efficiency over current redactable blockchains. For future work, we will focus on providing more comprehensive privacy protection (e.g., data privacy) and richer functionalities (such as accountability and revocation) in decentralized IoT systems. In addition, we will also focus on achieving richer data analytic mechanisms by combining various machine learning schemes.

## Figures and Tables

**Table 1:** Comparison between PFRB and existing redactable blockchain schemes.

	Fine-Grained	Decentralization	Policy-Hidden
AMVA17 [13]	✕	✕	✕
DSSS19 [16]	✓	✕	✕
DMT19 [14]	✕	✓	✕
TLL20 [24]	✓	✕	✕
PVM21 [25]	✓	✕	✕
JSZ21 [26]	✕	✕	✕
XNMX21 [27]	✓	✕	✕
XNMX22 [15]	✓	✕	✕
RBDS22 [18]	✓	✓	✕
Ours	✓	✓	✓

**Table 2:** Notations used in PFRB.

Notations	Descriptions	Notations	Descriptions
$\left(p,G,G_{0},e,g\right)$	description of bilinear group	λ	security parameter
(n,p,q,e,d)	description of RSA parameter	$H_{ID,G},H_{U,G},$ $H_{0},H_{b,Z_{p}}$	description of hash function
e(g,g)	description of the bilinear function	$pp_{s}$	public parameter
$pk_{s}$	public key	$sk_{s}$	secret key
msk	master secret key	ρ	an attribute set of modifier
$dp_{\rho }$	the decryption key	$\Delta_{i,R_{j}}$	Lagrange coefficient of ρ
id	global identifier	$sk_{id}$	modifier’s secret key
σ	modifier’s signature	θ	an authority
$\left(pk_{\theta },sk_{\theta }\right)$	authority’s own public key the secret key	*A*	an attribute
$sk_{id,A}$	the secret key of the modifier’s attribute *A*	$sk^{etd}$	the secret component of the ephemeral trapdoor
*R*	the target receiver of the transaction	$pk^{etd}$	public component of an ephemeral trapdoor
